# The global summit on the efficacy and effectiveness of spinal manipulative therapy for the prevention and treatment of non-musculoskeletal disorders: a systematic review of the literature

**DOI:** 10.1186/s12998-021-00362-9

**Published:** 2021-02-17

**Authors:** Pierre Côté, Jan Hartvigsen, Iben Axén, Charlotte Leboeuf-Yde, Melissa Corso, Heather Shearer, Jessica Wong, Andrée-Anne Marchand, J. David Cassidy, Simon French, Gregory N. Kawchuk, Silvano Mior, Erik Poulsen, John Srbely, Carlo Ammendolia, Marc-André Blanchette, Jason W. Busse, André Bussières, Carolina Cancelliere, Henrik Wulff Christensen, Diana De Carvalho, Katie De Luca, Alister Du Rose, Andreas Eklund, Roger Engel, Guillaume Goncalves, Jeffrey Hebert, Cesar A. Hincapié, Maria Hondras, Amanda Kimpton, Henrik Hein Lauridsen, Stanley Innes, Anne-Laure Meyer, David Newell, Søren O’Neill, Isabelle Pagé, Steven Passmore, Stephen M. Perle, Jeffrey Quon, Mana Rezai, Maja Stupar, Michael Swain, Andrew Vitello, Kenneth Weber, Kenneth J. Young, Hainan Yu

**Affiliations:** 1 Faculty of Health Sciences, Ontario Tech University, Oshawa, Canada; 2 Centre for Disability Prevention and Rehabilitation at Ontario Tech University and CMCC, Oshawa, Canada; 3 Division of Epidemiology, Dalla Lana School of Public Health, University of Toronto, Toronto, Canada; 4 IHPME, Dalla Lana School of Public Health, University of Toronto, Toronto, Canada; 5 Department of Sports Science and Clinical Biomechanics, University of Southern Denmark, Odense, Denmark; 6 Nordic Institute of Chiropractic and Clinical Biomechanics, Odense, Denmark; 7 Intervention & Implementation Research for Worker Health, Institute of Environmental Medicine, Karolinska Institutet, Stockholm, Sweden; 8 ELIB - et liv i bevegelse, Oslo, Norway; 9 Department for Regional Health Research, University of Southern Denmark, Odense, Denmark; 10 Department de Chiropractique, Université du Québec à Trois-Rivières, Trois-Rivières, Canada; 11 Department of Chiropractic, Faculty of Science and Engineering, Macquarie University, Sydney, Australia; 12 Department of Physical Therapy, Faculty of Rehabilitation Medicine, University of Alberta, Edmonton, Canada; 13 Canadian Memorial Chiropractic College, Toronto, Canada; 14 Department of Human Health & Nutritional Sciences, University of Guelph, Guelph, Canada; 15 Rebecca MacDonald Centre, Mount Sinai Hospital, Toronto, Canada; 16 Department of Health Research Methods, Evidence & Impact, Faculty of Health Sciences, McMaster University, Hamilton, Canada; 17 School of Physical & Occupational Therapy, McGill University, Montreal, Canada; 18 Faculty of Medicine, Memorial University of Newfoundland, St. John’s, Canada; 19 Faculty of Life Sciences and Education University of South Wales, Cardiff, UK; 20 Institut Franco-Européen de Chiropraxie, Ivry-Sur-Seine, France; 21 Faculty of Kinesiology, University of New Brunswick, Fredericton, Canada; 22 Department of Chiropractic Medicine, Faculty of Medicine, University of Zurich & Balgrist University Hospital, Zurich, Switzerland; 23 Department of Anesthesiology, University of Kansas Medical Center, Kansas City, USA; 24 RMIT University, Melbourne, Australia; 25 College of Science, Health, Engineering and Education, Murdoch University, Murdoch, Australia; 26 AECC University College, Bournemouth, UK; 27 Spine Center of Southern Denmark, University Hospital of Southern Denmark, Middelfart, Denmark; 28 Faculty of Kinesiology & Recreation Management University of Manitoba, Winnipeg, Canada; 29 School of Chiropractic, University of Bridgeport, Bridgeport, USA; 30 School of Population and Public Health, Faculty of Medicine, University of British Columbia, Vancouver, Canada; 31 School of Health, Medical and Applied Sciences, CQ University, Sydney, Australia; 32 Stanford University School of Medicine, Stanford University, Stanford, USA; 33 School of Sport and Health Sciences, University of Central Lancashire, Preston, England

**Keywords:** Spinal manipulation, Mobilization, Effectiveness, Efficacy, Systematic review, Non-musculoskeletal, Chiropractic

## Abstract

**Background:**

A small proportion of chiropractors, osteopaths, and other manual medicine providers use spinal manipulative therapy (SMT) to manage non-musculoskeletal disorders. However, the efficacy and effectiveness of these interventions to prevent or treat non-musculoskeletal disorders remain controversial.

**Objectives:**

We convened a Global Summit of international scientists to conduct a systematic review of the literature to determine the efficacy and effectiveness of SMT for the primary, secondary and tertiary prevention of non-musculoskeletal disorders.

**Global summit:**

The Global Summit took place on September 14–15, 2019 in Toronto, Canada. It was attended by 50 researchers from 8 countries and 28 observers from 18 chiropractic organizations. At the summit, participants critically appraised the literature and synthesized the evidence.

**Systematic review of the literature:**

We searched MEDLINE, Embase, the Cochrane Central Register of Controlled Trials, the Cumulative Index to Nursing and Allied Health, and the Index to Chiropractic Literature from inception to May 15, 2019 using subject headings specific to each database and free text words relevant to manipulation/manual therapy, effectiveness, prevention, treatment, and non-musculoskeletal disorders. Eligible for review were randomized controlled trials published in English. The methodological quality of eligible studies was assessed independently by reviewers using the Scottish Intercollegiate Guidelines Network (SIGN) criteria for randomized controlled trials. We synthesized the evidence from articles with high or acceptable methodological quality according to the Synthesis without Meta-Analysis (SWiM) Guideline. The final risk of bias and evidence tables were reviewed by researchers who attended the Global Summit and 75% (38/50) had to approve the content to reach consensus.

**Results:**

We retrieved 4997 citations, removed 1123 duplicates and screened 3874 citations. Of those, the eligibility of 32 articles was evaluated at the Global Summit and 16 articles were included in our systematic review. Our synthesis included six randomized controlled trials with acceptable or high methodological quality (reported in seven articles). These trials investigated the efficacy or effectiveness of SMT for the management of infantile colic, childhood asthma, hypertension, primary dysmenorrhea, and migraine. None of the trials evaluated the effectiveness of SMT in preventing the occurrence of non-musculoskeletal disorders. Consensus was reached on the content of all risk of bias and evidence tables. All randomized controlled trials with high or acceptable quality found that SMT was not superior to sham interventions for the treatment of these non-musculoskeletal disorders. Six of 50 participants (12%) in the Global Summit did not approve the final report.

**Conclusion:**

Our systematic review included six randomized clinical trials (534 participants) of acceptable or high quality investigating the efficacy or effectiveness of SMT for the treatment of non-musculoskeletal disorders. We found no evidence of an effect of SMT for the management of non-musculoskeletal disorders including infantile colic, childhood asthma, hypertension, primary dysmenorrhea, and migraine. This finding challenges the validity of the theory that treating spinal dysfunctions with SMT has a physiological effect on organs and their function. Governments, payers, regulators, educators, and clinicians should consider this evidence when developing policies about the use and reimbursement of SMT for non-musculoskeletal disorders.

**Supplementary Information:**

The online version contains supplementary material available at 10.1186/s12998-021-00362-9.

## Background

Some evidence-based clinical practice guidelines recommend that spinal manipulative therapy (SMT) be used alone, or in addition to other interventions for the management of back pain, neck pain, and headaches associated with neck pain [1–5]. Although health professionals who deliver SMT are primarily consulted for spinal pain, some patients are treated for non-musculoskeletal disorders [6–8]. Specifically, between 3 and 10% of patients who receive care from chiropractors and osteopaths are treated for non-musculoskeletal disorders [1, 6]. In relative terms, these figures suggest that only a small proportion of patients receive SMT for non-musculoskeletal disorders; however, in absolute terms, it indicates that a substantial number of patients globally receive such care every year.

The treatment of non-musculoskeletal disorders has a long tradition among chiropractors and osteopaths. This tradition is based on two foundational concepts. The first concept implies that spinal dysfunctions, or subluxations, can have a negative effect on the body’s innate ability to heal itself, and that these dysfunctions can be rectified through SMT [9–11]. The second concept proposes that spinal dysfunction can negatively impact the autonomic nervous system, which in turn may cause disease including organ dysfunctions [10–12]. Some argue that they may influence the autonomic nervous system and thereby improve physiologic function by correcting spinal dysfunctions through SMT [13, 14]. Many chiropractors do not endorse this thinking and use an evidence-based approach to clinical care [15–17].

Laboratory studies of physiological mechanisms report that certain types of manual therapies can indeed affect body functions, such as heart rate variability or inflammatory cytokines in healthy individuals [18, 19], thus supporting the notion that SMT can be used to treat non-musculoskeletal disorders. However, two systematic reviews suggest that such effects, if they occur, are short-lasting and without clinical consequences [13, 20]. Moreover, a recent randomized controlled trial which compared SMT to a successful sham control found no such effect [21]. Although essential to the understanding of physiological mechanisms of action of interventions, laboratory experiments alone have not provided a mechanistic understanding of these hypotheses nor provided evidence of clinical efficacy or effectiveness [22]. Therefore, as emphasized by Bialosky et al. [23], the hypothesized causal chain between SMT, the autonomic nervous system, and clinical outcomes remains hypothetical and has yet to be established.

Some clinicians and patients report favorable outcomes when SMT is used to treat a variety of non-musculoskeletal complaints, such as allergies, breathing problems, digestive problems, and tinnitus [24]. Moreover, case reports suggest that SMT may benefit patients who consult for conditions such as bedwetting [25, 26], multiple sclerosis [27], autism spectrum disorder [28], and ischemic stroke [29]. However, it is important to note that observations, including case-reports that shape clinical experience may be misleading for several reasons [30]. First, it is possible that the observed improvement is due to the natural course of the disease rather than the treatment that has been delivered. Second, contextual effects associated with the treatment may account for the reported improvements, rather than the treatment itself [19, 22, 31]. Moreover, whenever a treatment is provided, the patient may have expectations of the outcome, positive or negative, and it is well known that positive expectations of recovery are associated with favorable health outcomes [32]. Fourth, it is possible that the observed changes are due to concurrent treatments [33]. Finally, the observed improvement can be due to regression to the mean, whereby patients with more severe symptoms tend to show greater levels of improvements independently of the treatment they receive [34–36]. Consequently, RCTs are necessary to determine whether the benefits noticed in clinical practice and reported in case reports and case series are due to the proposed mechanisms of SMT or if they can be explained by other factors [33]. Without rigorously conducted RCTs, clinicians and patients may assume that SMT is more or even less effective than it is.

Several previous reviews have evaluated the efficacy and effectiveness of SMT for non-musculoskeletal disorders [20, 37–41]. Overall, these reviews found no strong evidence for the benefit of such treatment regardless of their scope, definitions of SMT, search strategies, and review methodology [20, 37–41]. Interestingly, these previous reviews have not had an obvious impact on health care and clinical policies, at least not within the chiropractic profession. We believe that this failed to occur because a broad-based consensus about the implications of this research has not yet been achieved within the chiropractic profession. Therefore, we convened a large group of international chiropractic researchers with different scientific backgrounds and expertise to anchor a new systematic review. Furthermore, to promote knowledge and understanding of our study to the chiropractic profession at large, we invited representatives from chiropractic associations and organisations to observe our research.

The purpose of our study was to systematically review the body of evidence on the efficacy and effectiveness of SMT for the prevention and treatment of non-musculoskeletal disorders. Based on the osteopathic and chiropractic theories described above [9–14], we assumed that the rationale for this treatment was the same across non-musculoskeletal disorders conditions; specifically, that treating spinal dysfunctions with SMT has a physiological effect on organs and their function. We addressed two main research questions for each of primary, secondary and tertiary prevention:

1. Compared to sham or placebo interventions, is spinal manipulation, spinal mobilization or spinal traction efficacious for the prevention or management of non-musculoskeletal disorders?
2. Compared to other interventions (including sham intervention when delivered in a pragmatic plan of management or no intervention), is spinal manipulation, spinal mobilization or spinal traction effective in the prevention or management of non-musculoskeletal disorders?

The primary target audience for our systematic review is policy makers (governments, insurers and regulators). We targeted policy makers because they are well positioned to facilitate the development of clinical practice guidelines and implement evidence-based policies that will serve and protect the public’s best interest. We also aim to provide educators, researchers and health care providers with the best evidence to inform their contribution to the policy development process.

## Context

### The global summit on the efficacy and effectiveness of spinal manipulation for the management of non-musculoskeletal disorders

The two research questions were the focus of the Global Summit on the Efficacy and Effectiveness of Spinal Manipulation for the Management of Non-musculoskeletal Disorders (Global Summit). The initiation of the Global Summit was prompted by international public concerns about chiropractic care for the management of non-musculoskeletal disorders [42–45].

#### Steering committee and writing team

The Global Summit was organized by a steering committee which included PC (chair), CLY, IA and JH. The steering committee developed the methodology for the systematic review, oversaw its conduct and implementation, led the evidence synthesis, and published the report. The steering committee was assisted in this work by a research assistant (MC). The final report was drafted by a writing team consisting of the steering committee and senior researchers with expertise and experience in evidence synthesis and scientific writing (JDC, SDF, GNK, SM, EP, JW). The writing team provided ongoing feedback and quality assurance to drafts of evidence tables and sections of the manuscript.

#### Participants

The Global Summit brought together researchers who were invited by the steering committee. Participants met the following criteria: 1) chiropractor with a PhD, or a researcher with a PhD (not a chiropractor) with research expertise in chiropractic; 2) actively involved in research (defined as having published at least 5 peer-reviewed papers over the past 5 years); and 3) appointed at an academic or educational institution. In addition, a small group of researchers who did not meet these criteria were invited. These included three chiropractors with a strong publication and scientific editorial record who did not have a PhD (SMP, JW and HS) and two early career researchers with an expertise within the area of chiropractic and pseudoscience (ALM, GG). Participants were invited by the Steering Committee using purposive and snowball sampling methods.

#### Pre-summit activities

From January 5, 2019 to September 13, 2019, the Steering Committee held regular meetings to organize the Global Summit. The presummit activities included: 1) identification and invitation of participants and observers; 2) design of the systematic review; 3) search of the literature; 4) submission of the review protocol to International Prospective Register of Systematic Reviews (PROSPERO); 5) development of the instruction manual for critical appraisal; 6) screening of articles; 7) creation of three review groups of researchers for studies related to primary, secondary and tertiary prevention; 8) critical appraisal of the literature (first round) conducted by each review group; and 9) preparation of structure and first draft of evidence tables.

#### Global summit meeting

On September 14–15, 2019, 50 researchers (31 males; 19 females) from eight countries met in Toronto, Canada for the Global Summit. Twenty-eight researchers were from North America, 14 from Europe and eight from Australia. There were no participants from Asia, Africa or South America. In addition, 28 observers from various chiropractic organizations and educational institutions from North America, Europe and Australia were present to observe the meeting. At the Global Summit, researchers worked in their pre-assigned review groups. Each group reviewed the eligibility of RCTs that were deemed to be relevant by participants prior to the Global Summit, rated and discussed the methodological quality of studies, and extracted data from eligible studies. This was one of four phases in the risk of bias assessment, as described in detail below.

#### Observers

The steering committee invited representatives from chiropractic organizations to observe the scientific deliberations during the Global Summit. The organizations represented at the Global Summit included 28 representatives from 18 international, national and provincial associations, regulators, one malpractice protective association, and one chiropractic college. We invited chiropractic organizations so that they could witness the scientific discussion and learn about the methods involved in the conduct of systematic reviews. There were no formal criteria to invite observers, but it followed a purposive process. Observers held separate meetings during the Global Summit to discuss the implications of the research. Although they were invited to observe the scientific discussion, they did not participate in or influence the scientific deliberations.

### Post-summit activities

Following the Global Summit, the steering committee ensured that all relevant studies were critically re-appraised using a standardized method and finalized the risk of bias assessment and evidence tables. The steering committee also led an online consensus process with all participants of the Global Summit, who were asked to review and approve/reject/modify the final risk of bias tables and evidence tables. These activities are discussed in detail below.

## Methods

### Protocol registration and reporting

We registered our systematic review with PROSPERO (CRD42019140194). We structured our report according to the Preferred Reporting Items for Systematic Reviews and Meta-Analysis (PRISMA) [46], the PRISMA Harms checklists [47] and synthesized the results according to the Synthesis without Meta-Analysis (SWiM) Guideline [48].

### Eligibility criteria

Studies eligible for our systematic review met the following inclusion criteria: 1) English language; 2) published from database inception to May 15, 2019 in a peer-reviewed journal; 3) investigated non-musculoskeletal disorders; 4) randomized controlled trial that investigated the efficacy or effectiveness of spinal manipulation, mobilization, or traction (all types including manual/assisted); 5) study population included all ages; 6) included at least one outcome that is specific to the non-musculoskeletal disorder under investigation and measured at the patient level; and 7) the number of randomized participants per group was ≥20.

We did not include the following study types: 1) guidelines, letters, editorials, commentaries, unpublished manuscripts, dissertations, government reports, books or book chapters, conference proceedings, meeting abstracts, lectures and addresses, consensus development statements, or guideline statements; 2) cadaveric or animal studies; 3) non-clinical studies (studies that aim to understand the physiological effects of spinal manipulation); 4) pilot studies aimed at demonstrating the feasibility of conducting an RCT; and 5) studies in which the effect of spinal manipulation, mobilization, or traction could not be isolated (e.g, studies where spinal manipulation was included in a multimodal program of care).

### Definitions of key concepts

#### Efficacy

Studies of efficacy are designed to investigate the benefits and adverse events of an intervention under ideal and highly controlled conditions. The preferred design for efficacy studies is the RCT using a sham or placebo group as a comparison [49].

#### Effectiveness

Studies of effectiveness seek to examine the outcomes of interventions under circumstances that more closely approximate a real-world setting. Effectiveness studies, therefore, typically use an RCT design, where the new treatment is compared to other interventions (including sham intervention when delivered in a pragmatic plan of management), such as the standard of practice for the patient population being studied [49]. In our review, we classified an RCT as an effectiveness trial if SMT was delivered according to a pragmatic plan of management regardless of the comparison group.

#### Non-musculoskeletal disorders

Disorders that are not related to the locomotor system, including those not related to disorders of muscles, bones, joints and associated tissues such as tendons and ligaments. These include but are not limited to asthma, stroke, migraine, dysmenorrhea and hypertension.

#### Primary prevention

Intervening to prevent disease or injury from ever occurring.

#### Secondary prevention

Intervening to cure or reduce the impact of a disease or injury that has already occurred.

#### Tertiary prevention

Intervening to improve the impact of a persistent illness or injury that has lasting effects.

#### Spinal manipulation

Manual therapy applied to the spine that involves a high velocity, low amplitude impulse or thrust applied at or near the end of a joint’s passive range of motion [50]. Spinal manipulation can be applied manually or with a mechanical device.

#### Spinal mobilization

Manual treatment applied to the spine that incorporates movements, within a joint’s passive range of motion [50, 51]. Spinal mobilization can be applied manually or with a mechanical device.

#### Spinal traction

Manual or mechanically assisted application of an intermittent or continuous distractive force [52, 53].

#### Spinal manipulative therapy

In this report, spinal manipulation, spinal mobilization and spinal traction are referred to collectively as “spinal manipulative therapy”.

### Information sources and search strategy

We developed our search strategy in consultation with a health sciences librarian from the Centre for Disability Prevention and Rehabilitation at Ontario Tech University and CMCC. A second librarian from the Canadian Memorial Chiropractic College reviewed the strategy to ensure accuracy using the Peer Review of Electronic Search Strategies (PRESS) checklist [54, 55]. We systematically searched MEDLINE U.S. National Library of Medicine (through Ovid Technologies Inc.), Embase, the Cochrane Central Register of Controlled Trials, Cumulative Index to Nursing and Allied Health (CINAHL, through EBSCOhost), and Index to Chiropractic Literature (ICL, Chiropractic Library Collaboration) from inception to May 15, 2019. Search terms consisted of subject headings specific to each database (e.g., MeSH in MEDLINE) and free text words relevant to manipulation/manual therapy, effectiveness, prevention, treatment, and non-musculoskeletal disorders (Additional file 1). We also asked participants to identify and submit any citations or articles that may be relevant to the literature review.

### Study selection

All articles retrieved through the literature searches were exported into EndNote X7.0.2 for reference management and tracking of the screening process. Four pairs of trained and experienced reviewers (HS, IA; SM, JH; CC, JW; AAM, PC) independently screened all potentially eligible articles in three phases. In phase one screening, titles and abstracts were reviewed and classified as relevant, possibly relevant or irrelevant according to the eligibility criteria. During phase two screening, the full text of possibly relevant articles was reviewed for final determination of eligibility. Pairs of reviewers discussed eligibility to reach consensus for both phases of screening. Finally, in phase three, the eligibility of studies identified in phase two was reviewed and adjudicated at the Global Summit by the primary, secondary and tertiary groups. In cases of disagreement between reviewers during phase one or phase two screening, a third independent reviewer (CLY) was consulted to achieve consensus.

### Data collection process and data items

We extracted the following descriptive variables from all relevant studies: First author’s name, year of publication, description of participants, case definition, health care setting where the study was conducted, sampling frame, total number of participants enrolled, treatment and control interventions (description, type of provider, number of participants at baseline), duration of follow-up; outcome measurement(s), results (between-group differences, risk ratio (RR) and 95% CI or *p*-values (when 95% CI were not reported or could not be computed)). The data were entered directly into evidence tables. Pairs of researchers extracted data during the Global Summit and independent reviewers validated the data extraction following the Global Summit. The steering committee subsequently validated the content of the evidence tables for completeness, accuracy and consistency of reporting. Finally, the content of the evidence tables was submitted to all participants for review and approval through an electronic survey. We used 75% agreement (38/50 participants) as the threshold for consensus.

### Risk of bias in individual studies

We critically appraised articles using the Scottish Intercollegiate Guidelines Network (SIGN) criteria for randomized controlled trials [56]. The SIGN criteria were selected by the steering committee for ease of use and relevance, and adapted for the purpose of our review by adding the following questions to the generic checklists:

- “The definition of the non-musculoskeletal condition is clear?” (Yes/No)
- “The participants are free from the non-musculoskeletal condition studied at the beginning of the study?” (Yes/No/Can’t say) (only for studies investigating primary prevention)
- “The spinal manipulative therapy (spinal manipulation, spinal mobilization, and spinal traction) intervention is described in sufficient detail?” (Yes/No)
- “The control intervention (if any) is described in sufficient detail?” (Yes/No)
- “The follow-up period is sufficient (long enough for the outcome to occur) to answer the research question?” (Yes/No/Can’t say)

In addition, we edited the following item (in the primary prevention form) to ensure that the measurement properties of the method used to identify the non-musculoskeletal condition were clearly captured. The item “Are all outcomes measured in a standard, valid and reliable way?” was split into two questions, “The non-musculoskeletal condition is measured in a reliable way” (Yes/No/Can’t say) and “The non-musculoskeletal condition is measured in a valid way” (Yes/No/Can’t say). Detailed notes accompanied the SIGN generic checklists, and these were also edited to match the purpose of this review.

The risk of bias assessment was informed by the items from the SIGN checklists that focused on methodological quality. All risk of bias assessments were conducted by two independent investigators who were unaware of each other’s ratings. The risk of bias items included: clarity of the research question, definition of the non-musculoskeletal condition, randomization procedure, blinding of participants, clinicians and investigators, description of manipulation and control interventions, outcome measurements, drop-outs, co-interventions, intention-to-treat analysis and follow-up period.

The risk of bias assessment was conducted in four sequential steps. Prior to the Global Summit, independent pairs of reviewers (within each of the primary, secondary and tertiary prevention review groups) critically appraised relevant RCTs to determine their methodological quality. At the Global Summit, the quality was discussed and agreed upon by the respective group. After the summit, all RCTs were critically appraised a third time by two independent experienced methodologists (CLY, JW, IA, SM, JH, PC) to ensure that the SIGN criteria were interpreted and applied in a similar manner across reviewers and review groups. Two participants (SF, EP) then performed quality assurance by reviewing all SIGN forms and risk of bias tables developed from the third round of reviews to ensure their accuracy and standard application.

A study was rated as low risk of bias (high or acceptable quality according to the SIGN methodology) if reviewers judged that potential sources of selection bias, information bias and confounding were minimal or acceptable [56]. In particular, reviewers focused on potential biases related to the randomization procedure, concealment of treatment allocation, blinding, administration of sham intervention, and attrition [57–61]. The presence of a validated sham procedure was considered particularly important.

Finally, all researchers involved in the systematic review of the literature reviewed the risk of bias tables and were asked to vote on the outcome of the critical appraisal through an electronic survey. We used 75% agreement (38/50 participants) as the threshold for consensus. One researcher (CLY) was not involved in the systematic review at the Global Summit but was, in case of problems, available as referee, to thereafter participate in the validation process and therefore also in the two voting sessions.

### Standardized metrics

We used RR and between-group difference in means to quantify the effect of interventions. We reported the intervention-specific incidence of adverse events. When these summary measurements were not reported in the published article, we used data reported in the paper to attempt to compute these statistics.

### Synthesis of results

We synthesized the evidence from acceptable or high-quality RCTs according to the SWiM Guideline and reported them in evidence tables [48]. We used two criteria to determine whether SMT was efficacious or effective. First, a study had to provide evidence that the null hypothesis was an unlikely hypothesis (*p* < 0.05) for the observed between-group difference in the primary outcome [62]. Second, if a difference was found, we determined whether the difference was clinically important. When available, we used standardized measurements (minimal clinically important difference [MCID]) to determine whether clinically important differences were reached in each trial. If the clinical importance of a statistically significant difference was not reported in the article, we planned to discuss the findings among Global Summit participants and reach consensus on its clinical importance. We used 75% agreement (38/50 participants) as the threshold for consensus.

We restricted our synthesis to RCTs with acceptable/high methodological quality because low/unacceptable quality trials are more likely to yield biased estimates of effect sizes [57–61]. To understand the impact of methodological quality on trial results, we contrasted results from methodologically acceptable studies with those from the unacceptable studies. The SWiM guideline was published after the registration of our protocol on PROSPERO [48]. Nevertheless, we adopted it to ensure that our evidence synthesis complied with the most current methods of reporting. We had initially planned to stratify the synthesis by primary prevention, secondary prevention and tertiary prevention. However, we revised this plan and synthesized the evidence by non-musculoskeletal disorder because there were no studies, and very few studies, to inform primary and tertiary prevention, respectively. We further synthesized the evidence, based on the study design (efficacy versus effectiveness).

We tabulated disease-specific outcomes as reported in the individual papers by comparing the outcomes for SMT to the outcomes for control interventions. These comparisons informed the development of an evidence statement for each non-musculoskeletal disorder. Because the studies were clinically heterogeneous, we did not assess for statistical heterogeneity of effects across studies.

We present our main results in a series of tables. First, we report our consensus methodological quality assessment in the risk of bias table. Second, the study characteristics and key study results are presented in the evidence table. Finally, we provide a succinct evidence table, which summarizes the key characteristics and results of all studies to facilitate the comparison of study results according to study quality. We examined the direction and magnitude of effect sizes across RCTs according to methodological quality by comparing studies rated as high/acceptable quality versus those rated as low/unacceptable quality.

We developed a consensus-based, narrative evidence statement for each non-musculoskeletal disorder. However, since the rationale for treatment is the same across conditions, these statements synthesize the evidence about the efficacy and effectiveness of SMT for the prevention and management of the specific non-musculoskeletal disorder in general, in accordance with our research questions.

### Publication bias and selective reporting

We did not assess publication bias. We checked reporting of outcomes for the acceptable- and high-quality trials against registered protocols by 1) scrutinizing the papers for mentioning of published or registered protocols; 2) searching for protocol papers in PubMed; and 3) accessing clinicaltrials.gov.

### Approval of the final manuscript and authorship

The final manuscript was submitted to all participants to the Global Summit for review. Participants were asked to vote electronically on whether they approved the final version of the paper and whether they wanted to co-author the published paper. Participants who declined authorship were asked to provide the reason for their decision. This process was repeated after the submitted manuscript had been reviewed by the journal.

## Results

### Study selection

**Fig. 1:**
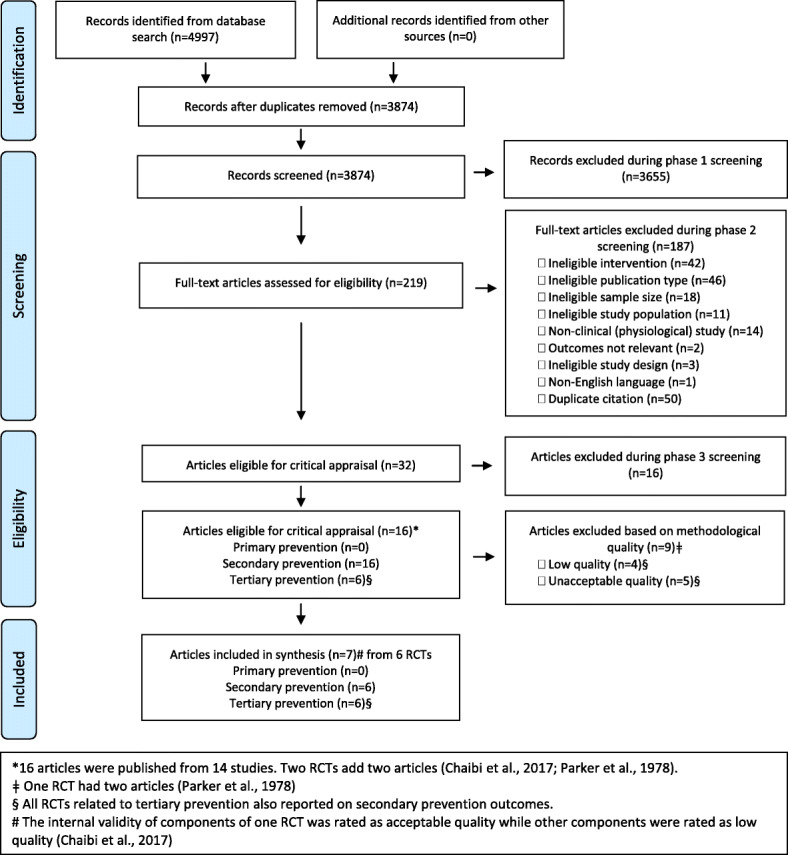
PRISMA Flow diagram

Our search retrieved 4997 citations (Fig. 1). No additional articles were submitted by participants. We removed 1123 duplicates and screened the titles and abstracts of 3874 citations (phase one screening). Of those, 219 citations were screened in phase two and the eligibility of 32 articles was reviewed at the Global Summit (phase three). The primary reasons for excluding 187 articles are presented in Fig. 1. Sixteen articles were excluded in phase three screening (Table 1) [18, 63–77]. Therefore, 16 articles (reporting on 14 RCTs) were included in the review and were critically appraised [37, 78–90]. We did not identify any RCTs related to primary prevention, 14 trials addressed secondary prevention, and six of the secondary prevention trials also included outcomes related to tertiary prevention. Of the acceptable and high-quality trials, one trial assessed efficacy [80] while five trials evaluated effectiveness [37, 81, 82, 84, 87].

**Table 1:** Primary reason for exclusion of RCTs in phase 3 screening

First author, Year	Population	Primary reason for ineligibility
Attali 2013 [63]^a^	Patients with irritable bowel syndrome	The sample size was < 20 per group.
Bevilaqua-Grossi 2016 [64]^a^	Patients with migraine and neck pain	SMT was part of a multimodal intervention. The effect of SMT could not be isolated. The multimodal intervention included medication plus a physiotherapy protocol diaphragm training, cervical mobilization and training, massage, myofascial release, trigger point therapy, passive stretching. The control group received medication alone.
Budgell 2006 [18]	Healthy adults	Experimental trial with physiological outcome (heart rate variability) not related to primary prevention of a non-musculoskeletal disorder
de Araujo 2018 [65]	Healthy asymptomatic individuals	Experimental trial with physiological outcome (heart rate variability) not related to primary prevention of a non-musculoskeletal disorder
Davidson 2018 [66]^a^	Patients with migraine	SMT was part of a multimodal intervention. The effect of SMT could not be isolated. The multimodal intervention included Maitland C0-C3 mobilization and Watson Headache Approach (exercise and advice). The control intervention was ‘wait list’.
Goertz 2002 [67]	Patients with high blood pressure or stage 1 hypertension	SMT was part of a multimodal intervention. The effect of SMT could not be isolated. The multimodal intervention included dietary modification, SMT and ultrasound, moist heat, soft-tissue massage. The control group received dietary modification alone.
Hensel 2013 [68]	Pregnant women at the 30th week of pregnancy	Experimental trial with physiological outcomes (arterial pressure and heart rate variability) not related to primary prevention of a non-musculoskeletal disorder
Holt 2016 [69]	Community-dwelling adults 65 years or older	Experimental trial with physiological/ biomechanical outcomes (joint position sense, choice stepping reaction time, postural stability, multisensory processing, health-related quality of life) not related to primary prevention of a non-musculoskeletal disorder
Jones 2015 [70]	Patients with dysfunctional breathing	SMT was part of a multimodal intervention. The effect of SMT could not be isolated. The multimodal intervention included respiratory physiotherapy plus Maitland mobilization, muscle energy technique, trigger point therapy, myofascial release, diaphragm and rib cage mobilization. The control group received respiratory physiotherapy alone.
Kachmar 2018 [71]	Patients with spastic forms of cerebral palsy	Experimental trial with outcomes (muscle spasticity, manual dexterity) not related to tertiary prevention of a non-musculoskeletal disorder
Nelson 1998 [72]^a^	Patients with migraine	SMT was part of a multimodal intervention. The effect of SMT could not be isolated. The multimodal intervention included SMT, massage and/or trigger point therapy with or without amitriptyline. The control intervention was amitriptyline alone.
Nielsen 1995 [73]	Patients with chronic asthma	The sample size was < 20 per group.
Noll 2000 [74]	Patients (≥ 60 years of age) hospitalized with acute pneumonia.	The osteopathic manipulative therapy did not include SMT.
Pizzolorusso 2014 [75]	Pre-term birth in infants	The osteopathic manipulative therapy did not include SMT.
Schwerla 2014 [76]^a^	Patients with primary dysmenorrhea	SMT was part of a multimodal intervention. The effect of SMT could not be isolated. The multimodal intervention included high velocity thrust, muscle energy technique, myofascial release, balanced ligamentous tension, visceral and cranial techniques. The control group was not treated.
Steele 2014 [77]	Patients enrolled in the study were between the ages of 6 months and 24 months with acute otitis media and an abnormal tympanogram.	SMT was part of a multimodal intervention. The effect of SMT could not be isolated. The multimodal intervention included combinations of Sacroiliac mobilization, myofascial release, balance ligamentous tension, suboccipital inhibition, venous sinus draining, occipital decompression, sphenobasilar decompression

*SMT* spinal manipulative therapy

^a^These studies were considered for both secondary and tertiary prevention

### Risk of bias within studies

Of the 14 included RCTs, three were rated as high quality [37, 81, 84], two were deemed to be of acceptable quality [80, 87], three were of low quality [79, 85, 89], and five were rated as unacceptable quality [78, 83, 86, 88, 90] (Table 2). The study by Chaibi et al. [82] received two quality ratings as the component of the trial comparing SMT to sham was rated to be of acceptable quality and sham was included in the evidence synthesis, whereas the component comparing SMT to the control intervention was rated as low quality because of the differentially high drop-out rate in the control group and that study was, therefore, not included in the evidence synthesis [82].

**Table 2:** Risk of bias table

Author, Year	2.1	2.2	2.3	2.4	2.5	2.6	2.7	2.8	2.9	2.10	2.11^a^	2.12^a^	2.13	2.14	2.15	3.5	Overall Ax
Goertz 2016 [81]	Y	Y	Y	Y	Y	Y	N	Y	Y	Y	Y	Y	SMT: 0% Sham: 1/27 = 4%	Y	N/A	Y	High Quality (++)
Hondras 1999 [37]	Y	Y	Y	Y	Y	Y	Y	Y	Y	CS	Y	Y	SMT: 2/69 = 3% Sham: 1/69 = 1%	Y	N/A	Y	High Quality (++)
Balon 1998 [84]	Y	Y	Y	Y	Y	Y	Y	Y	Y	Y	Y	Y	SMT: 7/45 = 16% Sham: 4/46 = 9%	CS	CS	Y	High Quality (++)
Olafsdottir 2001 [87]	Y	Y	CS	Y	Y	Y	Y	Y	Y	CS	Y	Y	SMT: 1/46 = 2% No SMT: 4/40 = 10%	Y	N/A	Y	Acceptable (+)
Ward 2015 [80]	Y	N	Y	Y	CS	Y	Y	Y	Y	Y	Y	Y	SMT: 0% No contact control: 0%	Y	N/A	Y	Acceptable (+)
Chaibi 2017 [82]	Y	Y	Y	Y	Y	Y	Y	Y	Y	Y	Y	Y	SMT: 8/35 = 23% Sham: 9/35 = 26% Usual pharmacological care: 14/34 = 41%	N	N/A	Y	Overall Acceptable (+) SMT vs. Control Low Quality (−)
Miller 2012 [89]	Y	N	Y	Y	CS	CS	Y	Y	Y	CS	Y	Y	Not blinded SMT: 7/33 = 21.2%^b^ Blinded SMT: 5/35 = 14.3%^b^ No SMT: 12/34 = 35.3%	CS	N/A	N	Low Quality (−)
Molins-Cubero 2014 [79]	Y	Y	Y	Y	N	Y	N	Y	Y	Y	Y	Y	SMT: 0% Sham: 0%	Y	N/A	N	Low Quality (−)
Wiberg 1999 [85]	Y	Y	Y	N	N	Y	Y	Y	Y	CS	Y	Y	SMT: 0% Advice: 9/25 = 36%	CS	N/A	N	Low Quality (−)
Qu 2012 [90]	Y	Y	CS	CS	N	CS	Y	Y	Y	CS	Y	Y	SMT: 0% Medication: 0%	Y	N/A	Y	Unacceptable (0)
Bakris 2007 [88]	N	N	Y	CS	CS	CS	Y	Y	Y	CS	Y	Y	SMT: 0/25 = 0% Sham: 1/25 = 4%	Y	N/A	Y	Unacceptable (0)
Tuchin 2000 [86]	Y	Y	N	N	N	CS	Y	Y	N	CS	CS	Y	SMT: max 4^c^ De-tuned IFT: max 4^c^	CS	N/A	N	Unacceptable (0)
Kokjohn 1992 [83]	Y	Y	CS	N	CS	CS	CS	Y	Y	CS	Y	Y	SMT: 1/24 = 4.2% Sham: 0/21 = 0%	Y	N/A	Y	Unacceptable (0)
Parker 1978 [78]	Y	Y	CS	N	N	CS	N	N	N	Y	Y	Y	SMT: at least 2/85 = 2.4%^c^ Mob: max 4/85 = 4.7%^c^	Y	CS	N	Unacceptable (0)

CS: can’t say; N: no; N/A: not applicable; Y: yes; ++: high quality; +: acceptable quality; −: low quality; 0: unacceptable quality/rejected

*IFT* interferential therapy, *Mob* mobilization

2.1 Research Question

2.2 Definition of non-MSK condition

2.3 Randomization

2.4 Concealment

2.5 Participant blinding

2.6 Investigator blinding

2.7 Groups are similar at start of trial

2.8 Description of manipulation intervention

2.9 Description of control intervention

2.10 Only difference between groups is the treatment

2.11 Reliability of outcome

2.12 Validity of outcome

2.13 Drop-out percentage

2.14 Subject analysis/Intention-to-treat

2.15 Comparable sites (if multiple)

3.5 Appropriate analysis

^a^Risk of bias table addresses primary outcome measures

^b^Participants were discharged but criteria for discharge were not outlined

^c^Did not outline which groups the drop-outs belonged to

Differences in the methodological quality between RCTs rated as high/acceptable quality and those rated as low/unacceptable quality were mainly related to the method of randomization, concealment of treatment allocation, successful blinding of participants (inability to identify the treatment), and blinding of outcome assessors and investigators (those who collected outcome data and investigators were unaware of the treatment received by participants) (Table 2).

Our qualitative synthesis therefore includes three high quality RCTs [37, 81, 84] and three RCTs of acceptable quality [80, 82, 87]. Of these, none evaluated the efficacy or effectiveness of SMT for the primary prevention of non-MSK disorders, six RCTs evaluated spinal manipulation for secondary prevention [37, 80, 82, 84, 87] and two studies evaluated spinal manipulation for tertiary prevention of non-musculoskeletal disorders [37, 84]. Both studies included in the tertiary prevention group were also included in the secondary prevention group.

Of the six trials of acceptable-or high-quality, two had been registered in *clinicaltrials.gov* [81, 82] and both reported outcomes in accordance with their protocols.

### Study characteristics

#### High and acceptable methodological quality

Six RCTs were rated as high or acceptable quality (Table 2). Of those, one investigated the efficacy of one session of diversified manipulation to T1-T4 for the management of adults with hypertension [80] (Table 3). The remaining five RCTs investigated the effectiveness of spinal manipulation for the management of: infants with colic [87]; children with asthma [84]; women with primary dysmenorrhea [37]; adults with hypertension [81]; and adults with migraines [82] (Table 3). These trials were clinically heterogeneous and therefore could not be pooled in a meta-analysis. Specifically, the trials included different populations, used different outcome measurements and were managed according to different therapeutic protocols.

**Table 3:** Evidence table for randomized controlled trials of high- and acceptable quality stratified by condition

**1st Author, Year, Study quality**	**Participants, Case definition, Setting,** **Number (n) enrolled**	**Interventions, Provider, Number (n) of subjects at baseline**	**Control, Provider, Number (n) of subjects at baseline**	**Follow-up**	**Outcome measurements**	**Results** **Mean (95%CI)**
***Asthma***						
Balon 1998 [84] Quality: High Quality	Children, 7 to 16 yrs.; physician diagnosed mild or moderate asthma > 1 yr, use bronchodilator at least three times weekly, and confirmed by lung function testing. Participants recruited through advertising. Private chiropractic clinics in Ontario, Canada. (*n* = 91)	SMT-HVLA determined and gentle soft-tissue therapy by chiropractor with usual medical care. 3x weekly for 4 weeks, 2x weekly for 4 weeks, then 1x weekly for 8 weeks, (range 20 to 36 Tx over 4-mths). Chiropractors with 5 years of clinical experience (*n* = 45)	Non-directional low amplitude, low velocity impulse to gluteals, scapulae and external occipital protuberance; and soft-tissue massage and gentle palpation; by chiropractor with usual medical care. 3x weekly for 4 weeks, 2x weekly for 4 weeks, then 1x weekly for 8 weeks, (range 20 to 36 Tx over 4-mths). Chiropractors with 5 years of clinical experience (*n* = 46)	2 and 4 mths	Primary outcome: morning PEF prior to use of bronchodilator and frequency of morning PEF of < 85% of baseline. Secondary outcomes: airway responsiveness; FEV1; daytime symptoms of asthma; need for inhaled beta-agonists; use of oral corticosteroids; Q of L; overall treatment satisfaction Adverse events	NS between group differences 2 mths (mean between group differences): PEF (% baseline): 2.1 (−3.9, 8.0) FEV_1_(liters): − 0.28 (− 0.61, 0.05) Overall Q of L: 0.29 (− 0.10, 0.69) Activity: 0.34 (− 0.13, 0.81) Symptoms: 0.29 (− 0.21, 0.78) Emotions: 0.26 (− 0.14, 0.66) Change in symptoms: *p* = 0.59 Use of Beta-agonists: *p* = 0.55 4 mths (mean between group differences): PEF: − 0.7 (− 6.7, 5.3) FEV_1_(liters): − 0.28 (− 0.61, 0.04) Overall Q of L: 0.32 (− 0.12, 0.75) Activity: 0.42 (− 0.10, 0.93) Symptoms: 0.15 (− 0.39, 0.69) Emotions: 0.29 (− 0.15, 0.73) Change in symptoms: *p* = 0.84 Use of Beta-agonists: *p* = 0.35 Between group differences in days with PEF < 85%: − 2.9 (− 11.1, 5.3) Mean satisfaction-Intervention: 6.22/7.0 Mean satisfaction-Control: 6.46/7.0 No adverse events, apart from exacerbations of asthma.
***Infantile Colic***						
Olafsdottir 2001 [87] Quality: Acceptable	Infants (born at term with a birth weight > 2.5 Kg; appropriate gain in weight, height and head circumference) recruited in Bergen, Norway from public health clinics the paediatric outpatient clinic at the University Hospital, general practitioners, chiropractors, and from direct referrals from parents who were informed about the project at the maternity units in Bergen and by the media from April 1998 to December 1999. Infantile colic defined as ≥3 h crying/day, 3 days per week for the last 3 weeks. (*n* = 100)	SMT and mobilization determined by treating chiropractor by areas of dysfunction identified by palpation; dysfunctional articulations manipulated and mobilized using light fingertip pressure. Counselling and support on feeding, baby care, and family interaction. 3 sessions with intervals of 2–5 days over 8 days by a licensed chiropractor (*n* = 46)	Infants held by a nurse for 10 min (the approximate time of treatment) after being partially undressed in a similar way as treated infants. Counselling and support on feeding, baby care, and family interaction. 3 sessions with intervals of 2–5 days over 8 days by a nurse (*n* = 40)	8–14 days post-intervention	Parent’s global perceived improvement (“getting worse”, “no improvement”, “some improvement”, “marked improvement”, “completely well”) Crying time (hours/day)	No Improvement in SMT group vs. control group (marked improvement or completely well) Relative Risk =0.97 (95% CI: 0.59, 1.60) Crying time (hours/day): SMT/mobilization vs. Control 1st visit: − 0.6 (− 1.47, 0.27) 2nd visit: − 0.5 (− 1.34, 0.37) 3rd visit: − 0.3 (− 1.17, 0.57)
***Hypertension***						
Goertz 2016 [81] Quality: High Quality	Adults (21–75 yrs), recruited from the community through targeted direct mailers, American Heart Association events, and press releases in Iowa, USA. Hypertension with systolic blood pressure ranging from 135 to 159 mmHg or diastolic blood pressure ranging from 85 to 99 mmHg and misalignment of either or both of the first 2 cervical spinal segments based on standardized radiography. Research clinic of the Palmer Center for Chiropractic Research, Davenport, IA (*n* = 51)	Toggle recoil consisting of HVLA thrust delivered to the C1 and/or C2 vertebra with participants in a side-lying position on the treatment table. 2 sessions/week over 6 weeks Chiropractors with > 5 years’ experience trained in toggle recoil SMT (*n* = 24)	Sham manipulation consisting of no thrust delivered to the participant’s head or neck. Delivered at 1st session and then for 4–8 visits at random intervals over 6 weeks Chiropractors with > 5 years’ experience trained in toggle recoil SMT (*n* = 27)	Immediately after intervention and 6 weeks	Primary outcome: Blood pressure Secondary outcome: SF–36 (Pain and General Health Sub-Scales), Perceived Stress Scale Adverse events	NS difference in blood pressure change between groups following the intervention. CRUDE: Mean difference change score 3 weeks: Systolic BP: − 0.3 (− 6.2, –5.3) Diastolic BP − 0.3 (− 4.0, –3.5) 6 weeks: Systolic BP: 3.5 (− 1.9, –8.9) Diastolic BP: 1.5 (− 2.1, –5.0) ADJUSTED (age, sex, BMI, baseline BP): Mean difference change score 3 weeks: Systolic BP: 0.9 (− 5.0, –6.9) Diastolic BP: 1.0 (− 2.8, –4.9) 6 weeks: Systolic BP: 4.8 (− 0.4, –10.0) Diastolic BP: 2.3 (− 1.2, –5.8) SF-36: Pain: Mean change (95% CI) SMT vs. Sham: 0.6 (− 2.18, 3.38) SF-36: General Health: Mean change (95% CI) SMT vs. Sham: − 1.1 (− 3.18, 0.98) Perceived Stress Scale Mean change (95% CI) SMT vs. Sham: 0.1 (− 1.70, 1.90) Adverse events included 4 related to study treatments: 3 headaches, 1 neck and upper thoracic pain. Three additional: foot numbness and tingling after a neck examination, fainting episode 24 h after treatment, mild nausea and vertigo at first treatment.
Ward 2015 [80] Quality: Acceptable	Adults (18–65 yrs), recruited via online advertisements and word-of-mouth in Texas, USA. Proof of high blood pressure (hypertension medications) or initial blood pressure reading > 140/90 mmHg. (*n* = 50)	Supine diversified anterior upper thoracic SMT to the T1–4 region with a HVLA thrust of the upper body of the chiropractor over the participant’s chest to achieve cavitation of the T1–4 segments of the thoracic spine. 1 session by chiropractor with 20 years of experience and 15 years of SMT technique teaching experience at Texas Chiropractic College (*n* = 25)	Participants’ arms folded across their chest for a few seconds and then the chiropractor unfolded the arms. 1 session by chiropractor with 20 years of experience and 15 years of SMT technique teaching experience at Texas Chiropractic College (n = 25)	1- and 10-min post-intervention	Bilateral blood pressure Arterial pressure Heart rate Adverse events not assessed	NS differences in mean blood pressure change between groups. Control vs SMT -Mean difference (95% CI) Right systolic BP 1 min: 3.4 (− 4.06, 10.86) 10 min: 2.9 (− 4.01, 9.81) Right diastolic BP 1 min: 2 (− 2.49, 6.49) 10 min: − 0.2 (− 4.69, 4.29) Left systolic BP 1 min: − 1.8 (− 8.67, 5.07) 10 min: 4 (− 3.39, 11.39) Left diastolic BP 1 min: − 0.6 (− 5.51, 4.31) 10 min: 1 (− 3.75, 5.75) Pulse pressure 1 min: 1.3 (− 3.84, 6.44) 10 min: 3.1 (− 1.51, 7.71) Mean arterial pressure 1 min: 2.5 (− 2.62, 7.62) 10 min: 0.9 (− 4.08, 5.88) Heart rate 1 min: 1.1 (− 2.97, 5.17) 10 min: 1 (− 3.12, 5.12)
***Dysmenorrhea***						
Hondras 1999 [37] Quality: High Quality	Women, 18–45 yrs.; sexually active, non-pregnant, good general health, regular menstrual cycles accompanied by moderate to severe pain; diagnosis of primary dysmenorrhea recruited through local advertisements in Chicago metropolitan newspapers. National College Chiropractic Center outpatient clinic, Chicago, USA. (*n* = 138)	SMT-HVLA > 750 N to all clinically relevant levels from T10-L5 and sacroiliac joints, bilaterally. 3x/week beginning the week before expected onset of menstruation for next two cycles (cycles 3 and 4) Chiropractors practicing at National College Chiropractic Center (*n* = 69)	LFM-high-velocity, short-lever, low amplitude thrust between 200 to 400 N to L2–3 vertebral by chiropractor Chiropractors practicing at National College Chiropractic Center (*n* = 69)	4 menstrual cycles	Primary outcome: Pain intensity (VAS) Secondary outcome: MDQ Adverse events	NS between group differences at any follow-up menstrual cycle for pain intensity (*p* = 0.65) or menstrual distress (*p* = 0.78) 2 women in the LFM group and 3 women in the SMT group reported soreness in the low back region 24–48 h following intervention at 1 visit.
***Migraine***						
Chaibi 2017 [82] Quality: Acceptable	Adults (18–70 yrs) recruited from Akershus University Hospital, general practitioners and media advertisements in Akershus and Oslo Counties, Norway. Migraine diagnosed according to the ICHD-II (ICHD, 2004) and with ≥ one migraine attack per month. Akershus University Hospital, Norway (*n* = 104)^a^	Gonstead method, specific contact, HVLA, short-lever SMT with no post-adjustment recoil that was directed to spinal biomechanical dysfunction (full spinal column approach) 12 sessions over 3 mths Experienced chiropractor (*n* = 34)	Sham SMT consisting of a broad, non-specific contact, low velocity, low amplitude sham push maneuver 12 sessions over 3 mths Experienced chiropractor (*n* = 34)	Immediately after treatment, 3, 6 and 12 mths.	Primary outcome: Number of migraine days per mth Secondary outcomes: migraine duration, migraine intensity and headache index, medicine consumption and adverse events	Significant differences in mean change in migraine days favoring sham treatments. No difference in secondary outcomes. Sham vs. CSMT Migraine days Post-treatment: − 1.6 (− 3.09, − 0.10) 3 months: − 1.7 (− 3.28, − 0.12) 6 months: − 0.8 (− 2.50, 0.90) 12 months: − 2.1(− 3.84, − 0.36) Duration Post-treatment: − 1.1 (− 3.19, 0.98) 3 months: − 1.2 (− 3.51, 1.10) 6 months: 2.0 (− 0.38, 4.38) 12 months: − 1.5 (− 4.05, 1.04) Intensity Post-treatment: − 0.1 (− 1.06, 0.86) 3 months: − 0.5 (− 1.49, 0.49) 6 months: 0.4 (− 0.77, 1.57) 12 months: − 1.1 (− 2.34, 0.14) Headache Index Post-treatment: − 170.4 (− 346.30, 5.50) 3 mths: − 143.4 (− 323.24, 35.45) 6 mths: − 115.2 (− 296.07, 65.67) 12 mths: − 232.9 (− 429.06, − 36.74) NS difference in medicine consumption Minor, transient adverse events [local tenderness and neck pain] were more commonly reported in CSMT (73/355) than sham SMT group (29/348). There were no severe or serious AEs reported.

*FEV1* forced expiratory volume in 1 s, *HVLA* high velocity, low amplitude, *mths* months, *NS* non-significant, *PEF* peak expiratory flow, *Q of L* quality of life, *SMT* spinal manipulative therapy, *Tx* treatment, *yrs* years, *LFM* low force mimic, *MDQ* Moos’ menstrual distress questionnaire, *VAS* visual analog scale, *AE* adverse events

^a^Results only reported from intervention (SMT) and sham group

#### Low and unacceptable methodological quality

Nine RCTs were rated as low or unacceptable quality (Table 1). Two of these were conducted in infants with colic [85, 89], two in women with dysmenorrhea [79, 83], one in adults with hypertension [88], one in adults with irritable bowel syndrome [90], and three in adults with migraines [78, 82, 86] (Table 4). Two studies evaluated the efficacy of spinal manipulation [79, 83] and seven evaluated effectiveness of spinal manipulation [78, 82, 85, 86, 88–90].

**Table 4:** Summary of findings for studies of acceptable quality

Author, Year	Origin of study sample	Study population	Interventions	Time of follow-up	Outcome variables	SMT superior to control
**Asthma**						
Balon, 1998 [84]	Chiropractic patients	Children (7–16 years) with mild or moderate asthma	SMT vs. Sham	2 and 4 months	FEV1 PEF Quality of life	No
**Infantile Colic**						
Olafsdottir, 2001 [87]	Public health care clinics, pediatric outpatient clinic at University hospital, general practitioners, chiropractors and direct referrals	Infants with colic	SMT vs. Sham	8–14 days after intervention	Parent’s global perceived improvement or crying time	No
**Hypertension**						
Goertz, 2016 [81]	Community in Iowa, USA	Adults with pre-hypertension or hypertension	SMT vs. Sham	Immediately and 6 weeks	Blood pressure	No
Ward, 2015 [80]	Community in Texas, USA	Adults with hypertension	SMT vs. Sham	Immediately and 10 min	Blood pressure, pulse pressure, mean arterial pressure and heart rate	No
**Dysmenorrhea**						
Hondras, 1999 [37]	Chiropractic patients	Women with primary dysmenorrhea	SMT vs. Sham	1 h	VAS or MMDQ	No
**Migraine**						
Chaibi, 2017 [82]	University hospital, general practitioners and community in Oslo Counties, Norway	Adults with migraine	SMT vs. Sham	Immediately, 3 months	Migraine days	No (Sham significantly superior to SMT)
				6 and 12 months	Migraine duration, intensity or headache index	No

Legend: *FEV1* forced expiratory volume in 1 s, *PEF* peak expiratory flow, *SMT* spinal manipulative therapy, *MDQ* Moos’ menstrual distress questionnaire, *mths* months, *NS* non-significant, *VAS* visual analog scale

### Evidence summary for the secondary and tertiary prevention of non-MSK disorders

#### Studies of high/acceptable quality

None of the six RCTs of high or acceptable quality demonstrated that SMT is efficacious or effective for the secondary or tertiary prevention of non-MSK disorders (Tables 3-4) and there were no studies on primary prevention.

##### Childhood asthma

One high quality RCT by Balon et al. [84] compared the outcome of spinal manipulation to that of simulated spinal manipulation for the management of mild or moderate asthma in individuals aged 7–16 years (Table 3). Both treatment groups received usual medical care. No statistically significant differences in morning peak expiratory volume were found between groups at the two- and four-months follow-ups. Similarly, there were no differences in secondary outcomes at follow-up (airway responsiveness, forced expiratory volume (FEV1), daytime symptoms of asthma, need for inhaled beta-agonists, use of oral corticosteroids, or quality of life). No adverse events were reported except exacerbations of asthma symptoms. This trial found that spinal manipulation is not effective for the management of mild or moderate asthma in individuals aged 7–16 years.

##### Infantile colic

One RCT of acceptable quality by Olafsdottir et al. [87] compared the outcome of spinal manipulation and mobilization using light fingertip pressure to the spine of an infant being held by a nurse for 10 min for the management of colic in infants aged 3 to 9 weeks (Table 3). Both groups also received parent counselling and support on feeding, baby care and family interactions. The authors found no difference in global improvement as perceived by parents or crying time at 8 to 14 days follow-up. This trial suggested that spinal manipulation and mobilization are not effective for the management of colic in infants aged 3 to 9 weeks. The authors did not report on adverse events.

##### Primary dysmenorrhea

In one high quality RCT, Hondras et al. [37] compared the outcome of high-velocity low-amplitude manipulation targeting the lower thoracic spine, lumbar spine and sacro-iliac joints to that of a low force mimic maneuver in females aged 18–45 years with primary dysmenorrhea (Table 3). The authors reported no difference in pain and prostaglandin levels in four subsequent menstrual cycles. Mild adverse events (transient post-treatment soreness in the low back) were reported by a few women in both groups. This RCT suggested that spinal manipulation is not effective for the management of primary dysmenorrhea in females aged 18–45 years.

##### Hypertension

One acceptable quality RCT [80] and one high quality RCT [81] informed the management of hypertension using spinal manipulation in adults. The first trial by Ward et al. evaluated the efficacy by comparing a supine diversified high-velocity low-amplitude manipulation to the T1–4 region to a sham procedure in adults between the ages of 18–65 with pre-hypertension or hypertension (Table 3) [80]. No differences in blood pressure, arterial pressure or heart rate were found between groups one- and 10-min post-treatment. These results agree with the findings of an effectiveness trial which compared toggle recoil thrust delivered to the C1-C2 region to sham manipulation in adults between the ages of 21–75 years with hypertension. In their RCT, Goertz et al. [81] found no differences between groups in blood pressure, health-related quality of life or perceived stress immediately after the intervention and at 6 weeks follow-up. Adverse events included three people with headaches and one with neck and upper thoracic pain [81]. These two RCTs suggested that spinal manipulation is neither efficacious nor effective for the management of hypertension in adults 18 years and older.

##### Migraine

An RCT of acceptable quality by Chaibi et al. [82] compared the outcomes of full-spine Gonstead high-velocity low-amplitude manipulation to sham manipulation for the management of adults with migraine headaches (Table 3). The results indicate that participants who received the sham manipulations had fewer migraine days per month during the one-year follow-up compared to the group receiving SMT (calculation based on published estimates, available from the authors on request). There were no differences between groups in migraine duration, intensity and medicine consumption at follow-up. Minor and transient adverse events (local tenderness and neck pain) were at least twice as common in the SMT group (73/355) as in the sham spinal manipulation group (29/348). This RCT suggested that spinal manipulation is not effective in the management of adults with migraine headaches.

#### Results of studies of low/unacceptable quality

All studies of low or unacceptable quality reported some positive results (Table 5). Eight RCTs rated as low or unacceptable quality reported at least some results supporting the efficacy [79, 83] or effectiveness of spinal manipulation [82, 85, 86, 88–90]. These studies reported on high blood pressure [88], infantile colic [85, 89], dysmenorrhoea [79, 83], irritable bowel syndrome [90], and migraine [82, 86]. A ninth study (of unacceptable quality) reported a significant improvement in migraine for its three study groups, but all groups received some type of manual therapy [78].

**Table 5:** Summary of findings for studies of unacceptable quality

Author, Year	Origin of study sample	Study population	Interventions	Time of follow-up	Outcome variables	Results from authors – SMT superior to control
**Infantile Colic**						
Miller, 2012 [89]	Chiropractic teaching clinic at Anglo-European College of Chiropractic	Infants with colic	SMT vs. Parent-blinded control	10 days or at discharge	24 h crying diary, Global improvement scale	Yes
Wiberg, 1999 [85]	Suburb of Copenhagen, Denmark	Infants with colic	SMT vs. Usual care	12 to 15 days	24 h crying diary, parents’ subjective evaluation of change	Yes
**Hypertension**						
Bakris, 2007 [88]	Unknown, USA	Adults with hypertension	SMT vs. Sham	8 weeks	Blood pressure	Yes
Molins-Cubero, 2014 [79]	Physiotherapy private practice; Madrid, Spain	Women with primary dysmenorrhea	SMT vs. Sham	Post-intervention	VAS	Yes
Kokjohn, 1992 [83]	Local community, local chiropractors or gynecologists; Illinois, USA	Women with primary dysmenorrhea	SMT vs. Sham	1-h post-intervention	VAS or MDQ	Yes
**Migraine**						
Chaibi, 2017 [82]	University hospital, general practitioners and community in Oslo Counties, Norway	Adults with migraine	SMT vs. Control	Immediately, 3 months	Migraine days, headache index	Yes
				6 and 12 months	Migraine duration or intensity	No
Tuchin, 2000 [86]	Radio and newspaper advertisements in Sydney region	Adults with migraine	SMT vs. Control	6 months	Frequency, duration, disability, use of medication	Yes
					Intensity, associated symptoms	No
Parker, 1978 [78]	Unknown; Australia	Adults with migraine	SMT vs. Mobilization	2 months	Duration, intensity, disability	No
**Irritable Bowel Syndrome**						
Qu, 2012 [90]	Outpatient department from Zhongda Hospital	Adults with irritable bowel syndrome	SMT vs. Drug	Post-intervention	VAS, Bowel symptom scale	Yes

Legend: *SMT* spinal manipulative therapy, *MDQ* Moos’ menstrual distress questionnaire, *mths* months, *NS* non-significant, *VAS* visual analog scale

### Review of risk of bias and evidence tables by global summit participants

The risk of bias table was approved by 98.0% (49/50) of participants (Table 2). Similarly, 98.0% (49/50) of participants approved the content of the evidence table (Table 3). The content of the evidence summary was approved by 100% (50/50) of participants for hypertension, 98% (48/49) for infantile colic, 94.0% (47/50) for dysmenorrhea, 94.0% (47/50) for asthma, and 90.0% (45/50) for migraine.

### Approval of the final manuscript and authorship

Eighty-eight percent of Global Summit participants approved the final paper and agreed to be co-authors. However, six participants declined authorship because they did not agree with the overall conclusion.

## Discussion

### Summary of findings

Our systematic review of the best available evidence suggests that SMT is not effective or efficacious for treating infantile colic, childhood asthma, hypertension, primary dysmenorrhea, or migraine. Collectively, the evidence from six high and acceptable quality RCTs casts doubt on the hypothesis that SMT is efficacious or effective for the management of non-musculoskeletal disorders [37, 80–82, 84, 87] and thereby also challenge the validity of the underlying theories relating to the subluxation and the autonomic nervous system [10, 11, 14].

### Previous literature

Our conclusions agree with several previous reviews. Clar et al., who comprehensively reviewed the literature on the clinical effectiveness of spinal manipulation for the management of musculoskeletal and non-musculoskeletal disorders, only found evidence for the effectiveness for the treatment of some musculoskeletal disorders [20]. Similarly, Goncalves et al., who reviewed evidence for spinal manipulation or chiropractic care as primary- or early secondary prevention for disease in general, failed to find any supportive evidence [42]. Ferrance and Miller, who reviewed the literature dealing with chiropractic diagnosis and management of non-musculoskeletal disorders in children broadly, including all types of studies, even case-reports, concluded that “The efficacy of chiropractic care in the treatment of non-musculoskeletal disorders has yet to be proven or disproven” [38]. Conversely, Kaminskyj et al. included case-reports, case-series, surveys, cohort studies and two RCTs and concluded that “it is obvious that some asthmatic patients may benefit from [chiropractic] treatment approach” but added that it should not replace traditional medical therapy [39]. Likewise, Pohlman and Holton-Brown reviewed 49 studies including commentaries, case-reports and case-series and concluded that possibly some children with otitis media may benefit from SMT [40]. Rist et al. reviewed RCTs of SMT as treatment for migraine headaches and included trials with high risk of bias where the effect of SMT could not be disentangled from the effect of co-interventions [91]. Nonetheless, they concluded that “SMT may be an effective therapeutic technique to reduce migraine days and pain intensity. However, given the methodological limitations to studies included [ …] we consider these results to be preliminary” [91]. Finally, Parnell Prevost et al. reviewed and critically appraised 50 studies of various designs dealing with manual therapy for a wide variety of pediatric conditions and concluded that the evidence was inconclusive but favorable for some non-musculoskeletal disorders including infantile colic (4 RCTs included), nocturnal enuresis (no RCTs included), sub-optimal infant breastfeeding (no RCTs included), respiratory, eyes, ears, nose and throat conditions (3 RCTs included) [43]. However, the review by Parnell Prevost et al. suffers from significant methodological limitations [92]. Our review adds to the literature because of four methodological differences between ours and some of the previous reviews. First, our research questions were different and focused on determining the efficacy and effectiveness of SMT for non-MSK disorders. Second, we restricted our search strategy to RCTs, which was necessary to assess efficacy and effectiveness. Third, we used different criteria to evaluate the methodological quality of RCTs. Finally, our evidence synthesis only included acceptable and high quality RCTs.

### Strengths and limitations

Our systematic review has several strengths. First, our research questions focused on determining the efficacy and effectiveness of SMT for both the prevention and management of non-musculoskeletal disorders. These questions required that we focus our review on evidence from high (*n* = 3) and acceptable (*n* = 3) quality randomized clinical trials. Second, the literature search was conducted by an expert librarian and independently reviewed by a second librarian to minimize errors. Third, our critical appraisal of the literature included four sequential steps to ensure that the risk of bias assessment was conducted in a transparent, standardized, and rigorous manner. Fourth, the evidence synthesis included only high and acceptable quality RCTs and was conducted according to the SWiM Guideline and reported in transparent evidence tables [48].

The findings of our review should, nevertheless, be interpreted in light of the following limitations. First, although our search method was thorough, it is possible that studies of high or acceptable quality were not retrieved because our literature search was restricted to the English language. However, it has been reported that excluding articles written in a language other than English does not lead to bias because most trials are published in the English literature [93–97]. Furthermore, authors included academics in the field with knowledge of German, Danish, Swedish, Norwegian, and French and none were aware of RCTs dealing with SMT published in those languages. This is supported by our search of the Index to Chiropractic Literature (ICL) which only identified RCTs published in English. Second, the critical appraisal of articles may vary among reviewers. However, our four-step approach to assessing risk of bias likely minimized this potential problem. Finally, publication bias may be present in this field of research. However, it is unlikely that publication bias compromised the validity of our results because studies most unlikely to be published are those that failed to obtain a ‘positive’ result. Further, all the low risk of bias RCTs included in our review show that SMT is not effective for the management of non-musculoskeletal disorders.

### Future review updates

Our findings, which are based on the best current evidence, may need to be modified with the publication of findings from new high-quality RCTs. Therefore, we recommend that our systematic review be updated every 2 to 3 years when new evidence becomes available. This is necessary to ensure that our findings are up to date with the most recent published literature. This is particularly important since our findings and conclusions are based on a limited number of high and acceptable quality trials, and only single trials for all but one conditions. Therefore, future trials can potentially alter our findings and conclusions. For example, we are aware of one ongoing RCT on the effectiveness of manipulation/mobilization for the management of infantile colic [98]. Once published, the quality of this trial should be evaluated, and its results integrated in an updated review. We recommend that governments, payers, regulators, educators and clinicians regularly adapt their policies and practices with new emerging evidence.

### Implications

The findings from the Global Summit call for the development and implementation of evidence-based policies regarding the use of SMT in the treatment of non-MSK disorders at several levels, as explained below. We anticipate that system-level polices will eventually impact clinical practice and change clinical behaviours. Policies should be based on the best available evidence with consideration of its strength and limitations.

i) *Implications for healthcare delivery systems and regulatory agencies*

Our systematic review highlights the need for healthcare delivery systems and regulatory agencies to consider the lack of evidence supporting the prevention and treatment of non-musculoskeletal disorders using SMT when developing policies.

ii) *Implications for educational institutions and educational regulators*

Educational institutions for the chiropractic, osteopathic and other manual medicine health professions have the opportunity to implement our findings into their curricula to train their students as modern, evidence-based clinicians. This will ensure that students’ future clinical activities are consistent with the best available evidence and viable within modern healthcare systems. Implementing our findings will require significant changes in curriculum in some institutions where there is a need to communicate to students when their teaching content is not supported by valid evidence. It is of utmost importance that educational institutions educate students to be competent consumers of research, thereby enabling the next generation of clinicians to differentiate high from low quality research. This should be a priority because, as we have demonstrated, sometimes very different conclusions can be drawn from high versus low quality research.

Educational regulators, who oversee the educational quality of practitioners of manual medicine, should also align their standards with best evidence and ensure consistency across educational institutions and, ultimately, of practitioners of manual medicine around the world. According to the Council for Higher Education Accreditation (CHEA) International Quality Group (CIQG) [99] this could be achieved by: 1) guiding institutions and organizations in developing capacity for academic quality; 2) advancing understanding of international quality assurance; and 3) providing research and policy direction [99].

Finally, the findings from the Global Summit should be incorporated into continuing education programs and disseminated to clinicians, and professional organizations should align their policies and communications with the current evidence.

iii) *Implications for clinical practice*

Our systematic review helps clinicians by providing them with necessary knowledge to deliver evidence-based care to their patients. Even though non-musculoskeletal disorders make up a small proportion of patients in chiropractic and osteopathic practice [6, 8], implementing our findings will require changes for some clinicians in the way they communicate and practice. It is important to emphasize that patients with non-musculoskeletal disorders might still benefit from seeing practitioners of SMT. First, because many people with non-musculoskeletal disorders have musculoskeletal comorbidities that significantly impact their overall health and well-being [100]. Alleviating pain and discomfort originating from the musculoskeletal system can be an important contribution to the care of people with multi-comorbidities. Second, contextual effects, associated with any clinical encounter can have important psychological and physical effects on patients. However, the best available evidence suggests that it is not the SMT that is responsible for the observed treatment outcomes [37, 80–82, 84, 87].

Importantly, clinicians need to be aware that low- and poor-quality studies can lead to deceptive results. This was the case in our review; all low-quality studies reported “good” results whereas high-quality studies all reported null results. Therefore, studies with poor methodological quality should not be used to inform clinical practice.

iv) *Implications for future research*

Although we found consistent evidence that SMT is not efficacious or effective for the management of non-MSK conditions, our conclusions are based on a limited number of high and acceptable quality RCTs. Therefore, more and better RCTs should be conducted if the management of non-musculoskeletal disorders with SMT is a priority for patients, clinicians and decision-makers. However, this will require that the preliminary research leading to the design and conduct of an RCT follows a sequential and logical approach, where adequate pre-trial data allows for the formulation of rational inclusion criteria, power calculations and interventions that have a clear biological target [101].

### Reflections about the global summit

Our research brought together an international group of researchers who used established methods to search, screen, critically appraise, and synthesize the literature. We ensured that our methods and deliberations were transparent by inviting chiropractic stakeholder organisations to attend and observe the Global Summit proceedings. The observers are, therefore, able to testify to the rigor and the transparency of the work conducted at the Global Summit proceedings. Including these observers in our process was important due to the considerable debate in the chiropractic and other manual medicine professions about the efficacy and effectiveness of SMT to prevent and treat non-MSK disorders.

The Global Summit was a unique and historic event. Never in the history of chiropractic or any manual medicine profession has such a large international group of active researchers collaborated to produce such a comprehensive scientific report. Representatives from professional organizations observed the scientific process and discussions, and they were able to interact with the scientists during breaks and provided all participants with the opportunity to discuss scientific, professional and political issues in an informal and friendly atmosphere. In spite of involving a large group of researchers and the complicated logistics of the Summit, we were able to strictly adhere to our pre-determined methods. It is also noteworthy that 88% of all researchers who attended the Global Summit agreed with the final conclusions of this comprehensive review. The six participants, who chose to abstain from authorship, did so because they did not agree that the overall conclusion represented the results of the review.

## Conclusions

Our systematic review included six randomized clinical trials (534 participants) of acceptable or high quality investigating the efficacy or effectiveness of SMT for the treatment of non-musculoskeletal disorders. We found no evidence of an effect of SMT for the management of non-musculoskeletal disorders including infantile colic, childhood asthma, hypertension, primary dysmenorrhea, and migraine. This finding challenges the validity of the theory that treating spinal dysfunctions with SMT has a physiological effect on organs and their function. Governments, payers, regulators, educators, and clinicians should consider this evidence when developing policies about the use and reimbursement of SMT for non-musculoskeletal disorders.

## Supplementary Information

**Additional file 1.**

## Data Availability

The datasets used and/or analyzed during the current study are available from the corresponding author on reasonable request.

## Abbreviations

MSK: Musculoskeletal
SMT: Spinal manipulative therapy
RCT: Randomized clinical trial
