# Correction to: The global summit on the efficacy and effectiveness of spinal manipulative therapy for the prevention and treatment of non-musculoskeletal disorders: a systematic review of the literature

**DOI:** 10.1186/s12998-021-00368-3

**Published:** 2021-03-08

**Authors:** Pierre Côté, Jan Hartvigsen, Iben Axén, Charlotte Leboeuf-Yde, Melissa Corso, Heather Shearer, Jessica Wong, Andrée-Anne Marchand, J. David Cassidy, Simon French, Gregory N. Kawchuk, Silvano Mior, Erik Poulsen, John Srbely, Carlo Ammendolia, Marc-André Blanchette, Jason W. Busse, André Bussières, Carolina Cancelliere, Henrik Wulff Christensen, Diana De Carvalho, Katie De Luca, Alister Du Rose, Andreas Eklund, Roger Engel, Guillaume Goncalves, Jeffrey Hebert, Cesar A. Hincapié, Maria Hondras, Amanda Kimpton, Henrik Hein Lauridsen, Stanley Innes, Anne-Laure Meyer, David Newell, Søren O’Neill, Isabelle Pagé, Steven Passmore, Stephen M. Perle, Jeffrey Quon, Mana Rezai, Maja Stupar, Michael Swain, Andrew Vitiello, Kenneth Weber, Kenneth J. Young, Hainan Yu

**Affiliations:** 1 Faculty of Health Sciences, Ontario Tech University, Oshawa, Canada; 2 Centre for Disability Prevention and Rehabilitation at Ontario Tech University and CMCC, Oshawa, Canada; 3 Division of Epidemiology, Dalla Lana School of Public Health, University of Toronto, Toronto, Canada; 4 IHPME, Dalla Lana School of Public Health, University of Toronto, Toronto, Canada; 5 Department of Sports Science and Clinical Biomechanics, University of Southern Denmark, Odense, Denmark; 6 Nordic Institute of Chiropractic and Clinical Biomechanics, Odense, Denmark; 7 Intervention & Implementation Research for Worker Health, Institute of Environmental Medicine, Karolinska Institutet, Stockholm, Sweden; 8 ELIB - et liv i bevegelse, Oslo, Norway; 9 Department for Regional Health Research, University of Southern Denmark, Odense, Denmark; 10 Department de Chiropractique, Université du Québec à Trois-Rivières, Trois-Rivières, Canada; 11 Department of Chiropractic, Faculty of Science and Engineering, Macquarie University, Sydney, Australia; 12 Department of Physical Therapy, Faculty of Rehabilitation Medicine, University of Alberta, Edmonton, Canada; 13 Canadian Memorial Chiropractic College, Toronto, Canada; 14 Department of Human Health & Nutritional Sciences, University of Guelph, Guelph, Canada; 15 Rebecca MacDonald Centre, Mount Sinai Hospital, Toronto, Canada; 16 Department of Health Research Methods, Evidence & Impact, Faculty of Health Sciences, McMaster University, Hamilton, Canada; 17 School of Physical & Occupational Therapy, McGill University, Montreal, Canada; 18 Faculty of Medicine, Memorial University of Newfoundland, St. John’s, Canada; 19 Faculty of Life Sciences and Education University of South Wales, Cardiff, UK; 20 Institut Franco-Européen de Chiropraxie, Ivry-Sur-Seine, France; 21 Faculty of Kinesiology, University of New Brunswick, Fredericton, Canada; 22 Department of Chiropractic Medicine, Faculty of Medicine, University of Zurich & Balgrist University Hospital, Zurich, Switzerland; 23 Department of Anesthesiology, University of Kansas Medical Center, Kansas City, USA; 24 RMIT University, Melbourne, Australia; 25 College of Science, Health, Engineering and Education, Murdoch University, Murdoch, Australia; 26 AECC University College, Bournemouth, UK; 27 Spine Center of Southern Denmark, University Hospital of Southern Denmark, Middelfart, Denmark; 28 Faculty of Kinesiology & Recreation Management University of Manitoba, Winnipeg, Canada; 29 School of Chiropractic, University of Bridgeport, Bridgeport, USA; 30 Faculty of Medicine, School of Population and Public Health, University of British Columbia, Vancouver, Canada; 31 School of Health, Medical and Applied Sciences, CQ University, Sydney, Australia; 32 Stanford University School of Medicine, Stanford University, Stanford, USA; 33 School of Sport and Health Sciences, University of Central Lancashire, Preston, England

**Correction to: Chiropr Man Therap 19, 348 (2021)**

**https://doi.org/10.1186/s12998-021-00362-9**

Following publication of the original article [1], we were notified of a typo in the last name of the 43rd author.

- Originally published name: Andrew Vitello
- Corrected name: Andrew Vitiello

The original article has been corrected.
